# The Ifakara Ambient Chamber Test (I-ACT) for Evaluation of Indoor Residual Sprays: A Non-Inferiority Test of Sylando^®^ 240SC and SumiShield^®^ 50WG

**DOI:** 10.3390/insects17030304

**Published:** 2026-03-11

**Authors:** Jane Johnson Machange, Ahmadi B. Mpelepele, Frank S. C. Tenywa, Mzee Pwagu, Dickson Kobe, Saphina H. Ngonyani, Dismas S. Kamande, Isaya Matanila, Ibrahim Kibwengo, Jason Moore, Joseph B. Muganga, Ritha Rex Kidyalla, Prisca A. Kweyamba, Susanne Stutz, James W. Austin, Sarah Jane Moore, Ummi Abdul Kibondo

**Affiliations:** 1 Vector Control Product Testing Unit, Department of Environmental Health and Ecological Science, Ifakara Health Institute, Bagamoyo P.O. Box 74, Tanzaniasmoore@ihi.or.tz (S.J.M.);; 2 Vector Biology Unit, Department of Epidemiology and Public Health, Swiss Tropical & Public Health Institute, Kreuzstrasse 2, 4123 Allschwil, Switzerland; 3 Faculty of Science, University of Basel, Petersplatz 1, 4001 Basel, Switzerland; 4 BASF SE, Professional & Specialty Solutions, Public Health, 67117 Limburgerhof, Germany; 5 BASF Corporation, Professional & Specialty Solutions, Public Health Global Development, Research Triangle Park, NC 27709-3528, USA; 6 The Nelson Mandela African Institute of Science and Technology (NM-AIST), Arusha P.O. Box 447, Tanzania

**Keywords:** Sylando, chlorfenapyr, SumiShield, clothianidin, Ifakara Ambient Chamber Test (I-ACT), insecticide, bioassay, indoor residual spray, IRS, *Aedes aegypti*, *Culex quinquefasciatus*, *Anopheles gambiae*, *Anopheles funestus*

## Abstract

New insecticides for indoor residual spraying (IRS) are being developed to manage resistance. Chlorfenapyr (Sylando^®^ 240SC), a pro-insecticide, is metabolized by active mosquitoes into the toxic metabolite tralopyril. This mode of action requires adapted “free flying” bioassays (because chlorfenapyr is converted to its toxic metabolite tralopyril when mosquitoes are metabolically active). A miniature-experimental hut (MEH) assay was developed within the Ifakara Ambient Chamber Test (I-ACT) with a rabbit as a host to measure residual efficacy under controlled conditions. Sylando^®^ 240SC was compared with SumiShield^®^ 50WG (clothianidin) for 12-month residual efficacy against malaria and arbovirus vectors. Residual activity was assessed on mud, wood and concrete with two huts per substrate treated with Sylando^®^ 240SC, one with SumiShield^®^ 50WG, and one with untreated control. Five replicates of 20 mosquitoes per strain (malaria vectors: pyrethroid-susceptible *Anopheles gambiae* and -resistant *An. arabiensis* and *An. funestus*; culicines *Aedes aegypti* and *Culex quinquefasciatus*) were exposed overnight at one-week post spraying and monthly thereafter. Multivariable mixed-effect logistic regression with binomial errors and log link function assessed non-inferiority with a 7% margin on mosquito mortality as the primary outcome for malaria vectors. Both products induced delayed mortality, with higher effects on malaria vectors than culicines. Across all substrates and malaria species combined over the full 12 months of observation, Sylando^®^ 240SC was non-inferior to SumiShield^®^ 50WG on mortality measured at 72 h (76% vs. 67%, OR = 0.86, 95% CI: 0.77–0.97) and 168 h (89% vs. 82%, OR = 0.74, 95% CI: 0.63–0.87). Sylando^®^ 240SC performed comparably to SumiShield^®^ 50WG, supporting its use as an IRS additional option. The new I-ACT mini-experimental-hut assay provides a practical tool for evaluating pro-insecticides. The importance of free-flight evaluation methods for pro-insecticides is discussed.

## Simple Summary

Alternative classes of insecticides for indoor residual spray (IRS) are needed for insecticide resistance management. Chlorfenapyr (Sylando^®^ 240SC) is a pro-insecticide and requires mosquitoes to be active to metabolize the parent molecule into its insecticidal metabolite tralopyril, a process that may take longer than other insecticidal modes of action. To evaluate its residual activity, a new laboratory assay was developed using a miniature experimental hut with a rabbit as a host inside the Ifakara Ambient Chamber Test (I-ACT). This study’s aim was to measure the residual efficacy of Sylando^®^ 240SC using malaria and dengue vectors. The entomological efficacy of Sylando^®^ 240SC was assessed against SumiShield^®^ 50WG over 12 months on mud, wood, and concrete surfaces using twelve mini-huts. Each substrate had two huts for Sylando^®^ 240SC, one for SumiShield^®^ 50WG and one negative control (water). The study evaluated the non-inferiority of Sylando^®^ 240SC against three malaria vectors (pyrethroid-susceptible *An. gambiae* and -resistant *An. arabiensis* and *An. funestus*) and their efficacy on non-malaria vectors (*Aedes aegypti* and *Cx. quinquefasciatus*). Sylando^®^ 240SC demonstrated comparable performance to SumiShield^®^ 50WG IRS, making it a potential candidate for indoor residual spraying (IRS) in vector control. Additionally, a new bioassay has been developed for evaluating pro-insecticides.

## 1. Introduction

**Figure 1:**
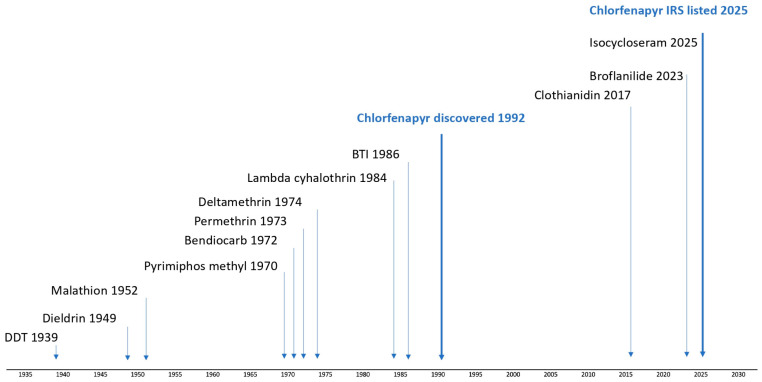
Timeline of discovery of insecticides for indoor residual spray.

Indoor residual spray (IRS) has historically relied on neurotoxic, fast-acting insecticides: dichlorodiphenyltrichloroethane (DDT) in the 1940s, dieldrin in the 1950s, organophosphates in the 1960s, pyrethroids in the 1970s, and carbamates in the 2000s (Figure 1). 

Consequently, IRS testing guidelines were designed around forced contact bioassays with short post-exposure observation periods [1]. These methods did not capture the efficacy of insecticides with delayed modes of action. Chlorfenapyr, for example, showed little effect in standard cone tests but killed free-flying mosquitoes in experimental hut trials [2], with higher mortality observed over longer holding times [3,4,5]. This highlighted the need for additional bioassays to evaluate slow-acting insecticides or non-neurotoxic insecticides, a need that remains critical for future product development.

For laboratory studies, the WHO cone test has been the standard assay for evaluating neurotoxic insecticides. For Sylando^®^ 240SC (chlorfenapyr), standard IRS bioassays underestimated efficacy because they limit mosquito metabolism through restricted movement, daytime testing, and short post-exposure observation periods. Bioactivation of chlorfenapyr to its toxic metabolite is maximal when mosquito metabolism is elevated [6]. In *Anopheles gambiae*, approximately 20% of its transcriptome (2800 of known genes) is regulated by circadian rhythms [7]. As a primarily nocturnal species, *Anopheles* exhibit peak flight metabolism at dusk and night [7,8], coinciding with host-seeking activity [9], and flight itself is supported by extremely high metabolic rates [10,11]. Experimental hut bioassays are generally regarded as the most representative proxy for IRS and insecticide treated net (ITN) performance against malaria vectors [12,13] as they allow free-flying mosquitoes to interact naturally with insecticide residues in the presence of a blood-host [14]. Notably, results from experimental hut trials [12] reflect the demonstrated clinical impact [15,16] of chlorfenapyr-treated ITNs.

A standardized assay using defined numbers of laboratory-reared mosquitoes of several species was still needed to verify the efficacy of IRS under controlled conditions. The Ifakara Ambient Chamber Test (I-ACT), originally developed to evaluate operationally aged ITNs [17], also demonstrated the potential of chlorfenapyr ITNs for malaria control [18]. Establishing a controlled IRS testing platform that allows overnight mosquito free-flight in the presence of a blood host, while incorporating delayed mortality measurements, offers the opportunity to reduce variability, increase statistical power, and cost-effectively generate evidence comparable to hut trials (Fairbanks, E. pers. comm). This study therefore aimed to assess whether Sylando^®^ 240SC was non-inferior to SumiShield^®^ 50WG when tested against malaria and dengue vectors in a small hut (mini-experimental hut) in the I-ACT with a rabbit as the host.

## 2. Materials and Methods

### 2.1. Study Design

A longitudinal semi-field evaluation of the entomological efficacy of Sylando^®^ 240SC (chlorfenapyr) compared with SumiShield^®^ 50WG was conducted over 12 months on mud, wood and concrete surfaces representative of those used in Tanzanian houses. Spraying took place in August 2022 and bioefficacy evaluations were performed monthly until September 2023.

The experiments were conducted in a semi-field tunnel called the Ifakara Ambient Chamber Test (I-ACT) [17]. The I-ACT allows free-flying mosquitoes to interact with treated surfaces under controlled conditions. The primary endpoint was mosquito mortality at 72 h as this is the standard holding time used for slow acting insecticides [19]. Mortality was also measured at 24 h intervals for up to 1 week (168 h) post exposure as a secondary endpoint. Blood feeding success was not considered a primary endpoint, since IRS generally functions by killing mosquitoes that are resting after feeding [20,21]. The primary analysis is based on the three Afrotropical vectors for 12 months of evaluation following the predefined analysis plan.

### 2.2. Study Area

This study was conducted at the Kingani site of the Ifakara Health Institute, Bagamoyo (70 km north of Dar es Salaam, Tanzania; 6°8′ S and 30°37′ E). The district receives 800–1000 mm of rainfall annually, with mean temperatures of 24–29 °C, and a mean relative humidity at 73% due to its coastal location. Insectaries and insecticide testing laboratories are located on site.

### 2.3. Ifakara Ambient Chamber Test for IRS Evaluation

**Figure 2:**
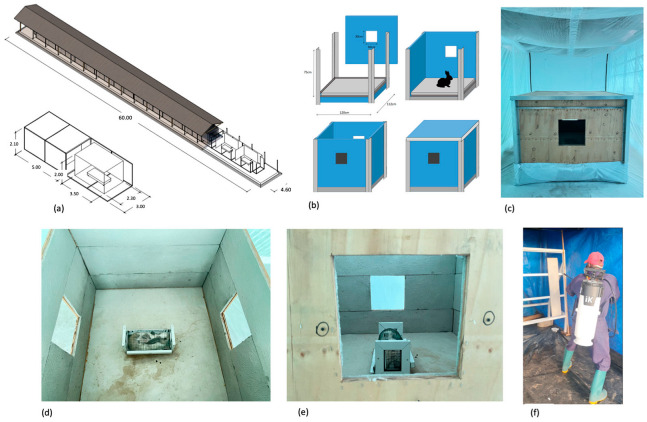
Mini-Experimental Hut (MEH) evaluation of indoor residual spray (IRS) in the Ifakara Ambient chamber test (I-ACT): MEH design and spraying procedures. (**a**) IACT, (**b**) mini hut, (**c**) mini hut inside the IACT, (**d**) inside view with a rabbit host, (**e**) window view of the mini hut, and (**f**) spraying panels.

The I-ACT has previously been used to evaluate IRS [22]. Twelve experimental compartments were fitted with mini-experimental huts (MEH, Figure 2a). Each MEH measures 120 × 112 × 75 cm and has square two openings (30 × 30 cm), allowing mosquito entry and exit (Figure 2b,c). The surface-area-to-volume ratio is 3.3, approximately double that of East African (1.3), West African (1.3), Ifakara (1.2), and Rapley (1.5) huts, maximizing the probability of mosquito contact with a treated surface. Panel features of each MEH are also designed to fit the standard for track sprayers [23]; however, manual spray was performed in this study.

### 2.4. Insecticides and Their Application

Sylando^®^ 240SC (chlorfenapyr, BASF, Ludwigshafen, Germany) was applied at 250 mg/m^2^. SumiShield^®^ 50WG (clothianidin 50% *w*/*w*, Sumitomo, Tokyo, Japan) was applied at 300 mg/m^2^. Both products are WHO PQ-listed [23]. Negative control (water) was included.

IRS products were mixed per manufacturer instructions and applied using IK Vector Control Super 7.5.1 backpack sprayers (Goizper, Antzuola, Spain) with a 1.5 bar control flow valve (CFV; discharge rate 30 mL/min). Separate sprayers were used for each treatment to avoid cross-contamination. Sprayers were calibrated, and gravimetric checks were performed by weighing depressurized tanks before and after spraying.

### 2.5. Substrate Preparation and Spraying Procedures

Substrate blocks (1–1.5 cm thick) of wood, concrete and mud were prepared. Plywood was cut into blocks for wood panels. Concrete blocks were made from 3333 g of cement Twiga Plus Portland Composite Cement (CEM II/B-M 42.5N; Tanzania Portland Cement Public Limited Company, Dar es Salaam, Tanzania) 1667 g of sieved sand, and 1 L of distilled water. Mud blocks were prepared from 1600 g of sieved soil, 2400 g of sand and 1 L of distilled water. Sand and mud were collected from the Wami River at the Ruvu bridge. Each mixture was stirred in separate plastic bowls for five minutes to ensure uniformity, applied to the rectangular surfaces using a trowel, and left to dry. Blocks were dried (mud: 1 week; concrete: 1 month after curing in distilled water for 3 days). Environmental conditions during curing were a median of 29.7 °C (interquartile range (IQR), 28.8–33.1) and 81.6% relative humidity (RH) (IQR 75.5–87.5). pH values were within the acceptable range: 7 for wood and mud; 9–10 for concrete.

Panels were sprayed outside the chambers and fitted to huts the same day. Protected spray tents prevented drift (Figure 2d). Application was standardized with metronomes to regulate spray speed and 45 cm lances to ensure correct distance. Whatman^®^ No 1. filter papers (Whatman International Ltd., Maidstone, UK) were attached to panels for spray quality monitoring. Four substrate samples and eight filter papers were sent to CRA-W, Belgium, for chemical verification.

### 2.6. Mosquitoes

Colony maintenance: *Anopheles* larvae were fed on Tetramin^®^ fish food (Tetra GmbH, Melle, Germany) and *Aedes* larvae on cat biscuits; adults received blood meals (cow blood from a membrane feeder) 3 to 6 days after emergence and 10% sugar solution ad libitum. Temperature and humidity within the insectary are maintained between 27 °C ± 2 °C and 70% ± 25% following MR4 guidelines [24].

The following strains were used:

(1) *Anopheles arabiensis* (Kingani), resistant to pyrethroids with mixed-function oxidases;
(2) *An. funestus* (Fumoz), resistant to pyrethroids with mixed-function oxidases;
(3) *An. gambiae s.s.* (Ifakara), fully pyrethroid-susceptible;
(4) *Culex quinquefasciatus* (Bagamoyo), resistant to pyrethroids with mixed-function oxidases;
(5) *Aedes aegypti* (Bagamoyo strain), fully pyrethroid-susceptible;
(6) *Ae. aegypti* (Kinondoni strain), resistant to pyrethroids and organophosphates.

Resistance Profile: The resistance profiles of the five laboratory strains were confirmed at the time of testing (Table S1) using both WHO tube assays [25] and gene expression analysis associated with metabolic insecticide resistance in *An. arabiensis*, *An. gambiae s.s.*, and *An. funestus*. The presence of target-site mutations was assessed using qPCR [26]. To investigate detoxification mechanisms, triplex RT-qPCR assays were conducted to evaluate the expression of seven cytochrome P450 detoxification genes (CYP6P3, CYP6M2, CYP9K1, CYP4G16, CYP6P4, CYP6P1, and CYP6Z1) and glutathione S-transferase epsilon 2 (GSTe2). Gene expression levels were compared against the ribosomal protein S7 (RPS7) reference gene. Following RNA extraction, RT-qPCR was performed to quantify gene expression. The detoxification genes exhibited low Ct values (<20) in the resistant *An. funestus* and *An. arabiensis* strains compared to the susceptible *An. gambiae* Ifakara strain. Ct values for the *RPS7* reference gene remained consistent across all samples, supporting the conclusion that the lower Ct values reflect a specific upregulation of detoxification gene transcripts. Relative expression patterns of all genes were analyzed using REST 2009 software v1.0 (Qiagen, Hilden, Germany).

### 2.7. Assay Conduct

A restrained rabbit served as bait in each MEH between 18.00 h to 09.00 h, covering host-seeking periods of *Anopheles* and *Culex* and early morning activity of *Ae. aegypti*. For each replicate, 20 nulliparous 3–5-day-old, sugar-fed, laboratory-reared mosquitoes of each strain were aspirated into cups by laboratory staff and released into each chamber outside the MEH by removing the net on the holding cups (Figure 2e,f). Morphologically identical *Anopheles* were dusted with fluorescent powder (Sigma Aldridge^®^, Burlington, MA, USA) to distinguish them.

After the allotted experimental time, all mosquitoes within each of the compartments were removed by prokopack aspirators and syphons (with HEPA filter). Surviving mosquitoes were placed in paper cups (fed and unfed are placed in different cups to allow measurement of pre- and post-prandial mortality if desired) and provided with 10% sucrose solution, and mortality was recorded at 24, 48, 72, 96, 120, 144 and 168 h.

Environmental conditions inside the I-ACT during the experiment were a median temperature of 26.2 °C ([IQR]: 25.1, 29.8) and 81.5% median RH (IQR: 77.9, 85.8). The holding room was maintained at a median temperature of 26.2 °C (IQR: 25.6, 27.2) and a median RH of 77.3% (IQR: 74.3, 85.2) at the time of the experiment (Figure S1).

### 2.8. Measures to Reduce Bias

Treatment allocation was randomized and coded with four-digit identifiers. Investigators recording outcomes were blinded until after database lock. Two huts per treatment arm improved precision. Huts were not rotated between chambers, to preserve sprayed surfaces, but chambers were structurally identical and any residual variance was adjusted in analysis.

### 2.9. Sample Size

Sample size was estimated using the precision of the confidence interval of the mean, assuming 80% mortality, 90% power and 99% confidence to detect a 99% confidence interval of 5% of the mean. Each replicate consisted of 20 mosquitoes per strain. With two huts per substrate per treatment arm and five nights per hut, 200 mosquitoes per strain per substrate per month were tested for Sylando^®^ 240SC. For positive and negative controls, 100 mosquitoes per strain per substrate per month were tested. In month 7, replication increased to eight nights to ensure adequate precision.

### 2.10. Statistical Analysis

Data were double-entered in Excel and exported into STATA 17 software (StataCorp LLC, College Station, TX, USA) for further cleaning and analysis. Mortality was summarized as arithmetic mean percentages with 95% CIs at 72 h and 168 h holding periods for each treatment and species presented.

Multivariable mixed-effect logistic regression with binomial error and log link function was used to compare Sylando^®^ 240SC to SumiShield^®^ 50WG. Treatment, chamber and day were fixed effects. For pooled analyses, substrate type was included as a fixed effect, and for analyses of multiple strains combined, mosquito strain (for malaria species) was also included as a fixed effect to increase statistical power and precision. Pooling malaria vectors was performed to estimate the overall efficacy of IRS, while species-specific analyses are presented in the Supplementary Material. The non-inferiority was assessed using the absolute difference in mosquito mortality between Sylando^®^ 240SC and SumiShield^®^ 50WG, with a predefined 7% margin based on WHO guidelines [27]. Effect estimates are reported as odds ratios (ORs) and 95% CIs over the 12-month period and interpreted following CONSORT guidance [28].

## 3. Results

### 3.1. Study Quality Check

Chemical analysis results of filter paper were within the target dose (Table 1).

**Figure 3:**
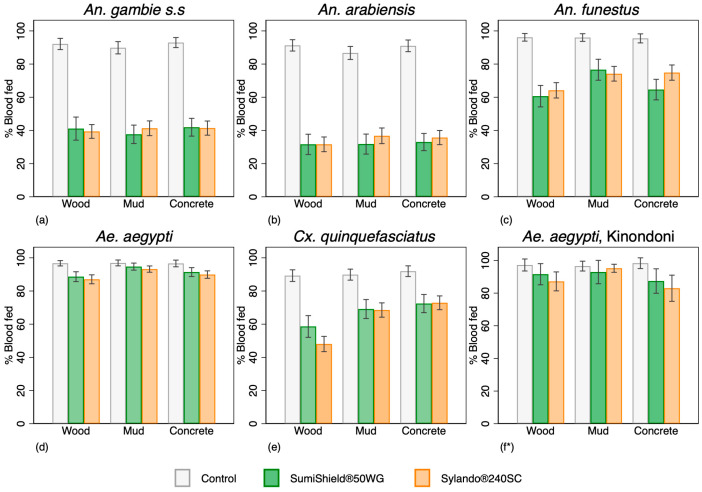
Overall blood-feeding success by species after exposure to Sylando^®^ 240SC and SumiShield^®^ 50WG in I-ACT over 12 months (pooled) of evaluation. (**a**,**d**) are susceptible to all insecticides and (**b**,**c**,**e**,**f**) are pyrethroid-resistant; * evaluation was performed from month 10 for pyrethroid-resistant *Aedes aegypti*.

Over the entire 12 months of evaluation, mosquito mortality at 168 h post spray in negative control huts for all strains and substrates was <15%, whilst blood feeding success was >88% (Figure 3).

### 3.2. Overall Mosquitoes’ Mortality

**Figure 4:**
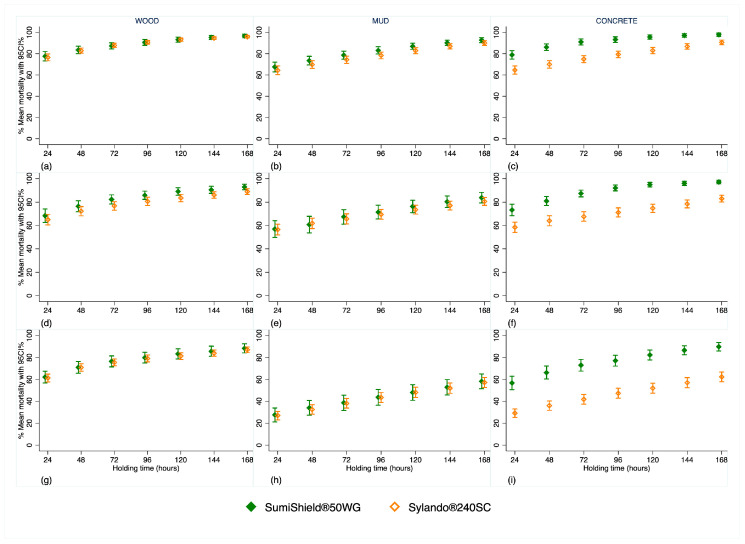
Overall mortality for 12 months of evaluation combined after exposure to Sylando^®^ 240SC and SumiShield^®^ 50WG in I-ACT. (**a**–**c**) Pyrethroid-susceptible strain *An. gambiae s.s.*; (**d**–**f**) pyrethroid-resistant *An. arabiensis*; (**g**–**i**) pyrethroid-resistant *An. funestus*.

Both products exhibited comparable performance, with mortality increasing over longer holding times across all substrates for each malaria vector species (Figure 4).

**Figure 5:**
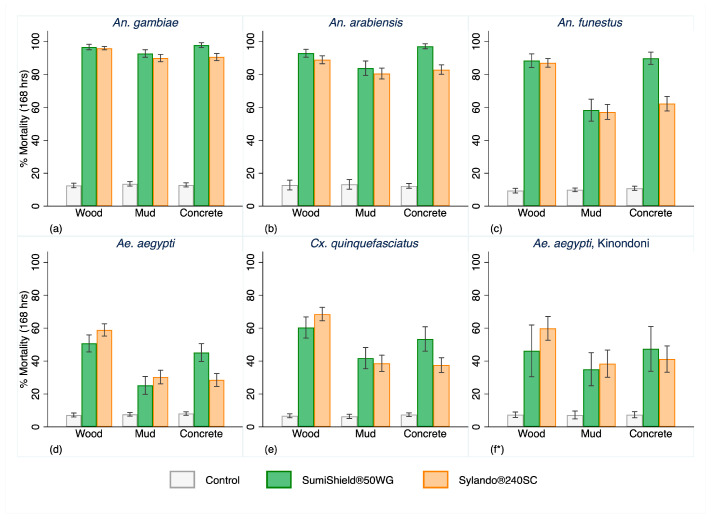
Overall mortality by species at 168 h post exposure to Sylando^®^ 240SC and SumiShield^®^ 50WG in I-ACT over 12 months (pooled) of evaluation. (**a**,**d**) are susceptible to all insecticides and (**b**,**c**,**e**,**f**) are pyrethroid-resistant; * evaluation was performed from month 10 for pyrethroid-resistant *Aedes aegypti*.

On wood and mud, mortality was almost identical between products, whereas SumiShield^®^ 50WG induced higher mortality on concrete (Figure 4 and Figure 5).

Malaria vectors consistently showed greater mortality than non-malaria vectors in both IRS treatment arms (Figure 5). After 168 h, mortality exceeded 80% for pyrethroid-susceptible *An. gambiae* and pyrethroid-resistant *An. arabiensis* on each substrate, and surpassed 60% for pyrethroid-resistant *An. funestus* (Figure 5). Full mortality data for each strain over the 12-month evaluation period, disaggregated by product and substrate, are provided in the Supplementary Materials (Figures S2 and S3).

### 3.3. Non-Inferiority Testing of Sylando^®^ 240SC and SumiShield^®^ 50WG; Afrotropical Vectors Combined

**Figure 6:**
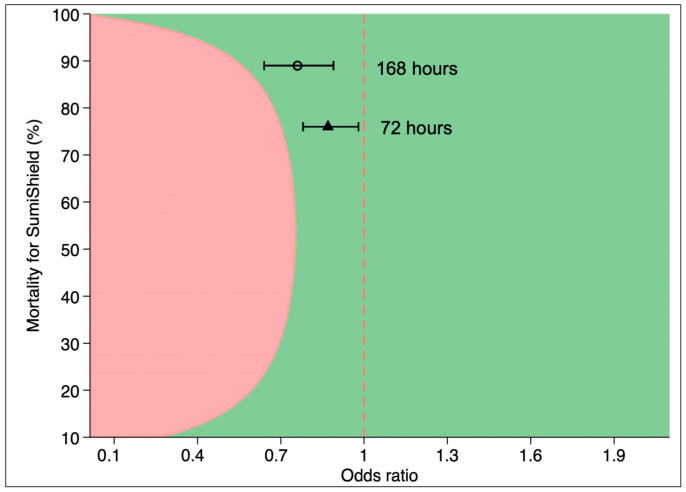
Non-inferiority of Sylando^®^ 240SC to SumiShield^®^ 50WG at 12 months with all substrates combined (concrete, wood and mud) for the three Afrotropical vector strains combined (pyrethroid-susceptible *An. gambiae s.s.*, pyrethroid-resistant *An. arabiensis* and -resistant *An. funestus*). The green region indicates an outcome favouring the candidate product (Sylando^®^ 240SC) and the pink region indicates outcomes favouring the active comparator (SumiShield^®^ 50WG).

Across all substrates combined and over the 12-month period, the combined mortality at 72 h for *An. gambiae* (pyrethroid-susceptible), *An. arabiensis* (pyrethroid-resistant) and *An. funestus* (pyrethroid-resistant) was 67% for Sylando^®^ 240SC compared to 76% in SumiShield^®^ 50WG [OR = 0.86, 95%CI: 0.77, 0.97]. By 168 h, mortality increased to 82% with Sylando^®^ 240SC and 89% with SumiShield^®^ 50WG [OR = 0.74, 95%CI: 0.63, 0.87]. Using a 7% non-inferiority margin, Sylando^®^ 240SC was demonstrated to be non-inferior to SumiShield^®^ 50WG at both 72 and 168 h holding times (Figure 6). Non-inferiority results for each species are presented in the Supplementary Materials (Table S2).

## 4. Discussion

### 4.1. Performance of Sylando^®^ 240SC and the Case of Chlorfenapyr

The Ifakara Ambient Chamber Test (I-ACT) clearly demonstrated that mortality significantly increased when mosquitoes were metabolically active (host-seeking), supporting the mode of action of chlorfenapyr as a pro-insecticide requiring oxidative activation to its toxic metabolite tralopyril [6]. This was also observed in earlier targeted IRS (TIRS) studies with *Aedes aegypti* in Mérida, Mexico, where delayed intoxication was recorded during extended observation of mosquitoes free-flying in experimental houses [29].

The delayed metabolic action of chlorfenapyr is challenging. Apparent “resistance” could be misattributed [30] if bioactivation is not complete, due to low temperature, incorrect photoperiod, or lack of mosquito movement [31]. This was evidenced by the significant disparity between forced-contact cone bioassays and the current study’s findings in the same facility. On mud substrate aged six months, chlorfenapyr caused 20.8% mortality in *An. arabiensis* and 4.5% in *An. funestus* at 72 h post exposure (Benson J. pers comm), while that in this study was 71% in *An. arabiensis* and 32% in *An. funestus*. In contrast, clothianidin, a neurotoxic insecticide showed high efficacy (98%) against *An. arabiensis* in this study, but more similar to the mortality measured in the cone assay at 120 h post exposure (83%) (Tenywa F. pers comm). Although mosquito movement was not recorded, these findings suggest that cone bioassays likely underestimate the full performance of pro-insecticide chemistries [5,18,32].

In addition, use of solvents that do not interact well with chlorfenapyr can lower observed mortality [30]. Mechanistically, chlorfenapyr is a pro-insecticide functioning as a protonophore. It accepts protons and facilitates their transport across the inner mitochondrial membrane, uncoupling oxidative phosphorylation. This process dissipates energy as heat, depletes adenosine triphosphate (ATP), and causes paralysis and death [33]. While altered expression of metabolic genes—particularly cytochrome P450s such as CYP6P1, CYP6P3, CYP6Z1 and CYP4G16—can affect pyrethroid susceptibility, these do not necessarily reduce chlorfenapyr activity and may even promote pro-insecticidal conversion [34]. However, detoxification via glutathione S-transferases (GSTs), notably GSTE2, could plausibly reduce susceptibility by conjugating halogenated compounds [35,36]. This underscores the need for nuanced interpretation of susceptibility changes rather than reliance on neurotoxic resistance markers, also highlighting the need for appropriate methods when testing efficacy or susceptibility to a pro-insecticide such as chlorfenapyr.

Currently, WHO PQT/VCP (World Health Organization Prequalification Team—Vector Control Products) lists several IRS insecticides including alpha-cypermethrin, bifenthrin, bendiocarb, clothianidin, deltamethrin, etofenprox, lambda-cyhalothrin, pirimiphos-methyl, broflanilide, isocycloseram, and chlorfenapyr. However, widespread pyrethroid resistance has led most National Malaria Control Programmes (NMCPs) to abandon older compounds. New chemistries are under exploration but are not yet listed. Given declining donor support, countries must diversify IRS portfolios to sustain efficacy through chemical rotation, especially as chlorfenapyr is widely used in ITNs. The use of Sylando^®^ 240SC for dengue control should also be considered, especially because of the long-lasting efficacy observed here in addition to positive results from other trials [29].

### 4.2. Variation in Performance Among Mosquito Species

Different mosquito species exhibit varying behaviours in the presence of intervention (e.g., resting, host seeking and feeding), which can influence the insecticidal efficacy. Lower mortality observed for *An. funestus* and non-malaria vectors may partly be explained by higher blood-feeding success relative to *An. gambiae* s.l., as blood-fed mosquitoes may retain greater energy reserves and therefore exhibit delayed mortality [37,38]. Previous studies have shown that blood feeding can enhance metabolic detoxification, increasing resistance intensity and allowing survival even at high insecticide doses [37].

*Anopheles* mosquitoes predominantly are nocturnal and typically rest for prolonged periods on treated wall surfaces after feeding, increasing their exposure to IRS-treated substrates [39]. This is particularly evident for *An. funestus*, a highly anthropophilic vector, which primarily bites during the late-night hours and rests indoors [40]. While the IACT-MEH conducted in this study effectively simulates these host-seeking and resting behaviours, it may not fully represent the behaviour of non-malaria vectors (*Culicines*), especially as data on entry and exit rates were not recorded. IRS is most effective against endophilic mosquitoes, whereas Dengue vectors in Tanzania are mostly exophilic [41,42]. Despite IRS being primarily optimized for malaria vector control in sub-Saharan Africa, the inclusion of *Culicine* species in this study was to explore potential added value for integrated vector control. Previous evidence from Mexico demonstrated that chlorfenapyr-based IRS significantly reduced *Ae. aegypti* populations and dengue transmission [29], suggesting that IRS may provide complementary benefits beyond malaria control. In addition, control of nuisance biting *Culex* will enhance community acceptance [43].

### 4.3. Monthly Fluctuations in Efficacy

Residual efficacy for both IRS products showed a non-linear pattern over the months. This fluctuation may reflect environmental influences [44], mosquito fitness [45] and changes in insecticide bioavailability [46]. While the bioavailability of chlorfenapyr may be temperature-dependent [31], no significant correlation was observed between mortality and temperature/humidity, both of which were within optimal ranges. The fluctuation could reflect changes in the surface bioavailability of insecticide deposits, mosquito fitness, and metabolic resistance, none of which were measured monthly. To account for monthly variation, day of testing was included as a fixed effect, and data were pooled across 12 months to provide an operational estimate of residual efficacy, adequate power, and precision for non-inferiority testing [19]. Programmatically, IRS products are selected based on overall residual activity to guide procurement and spray timing; accordingly, Sylando^®^ 240SC provided prolonged control and was non-inferior to SumiShield^®^ 50WG against Afrotropical vectors.

Mosquito physiological condition including age and body size may influence insecticide susceptibility [47]. While age-related susceptibility often decreases as mosquitoes mature, the consistent use of individuals aged 3 to 5 days in this study suggests that age was not the primary driver of the observed mortality fluctuations. The observed differences in mortality particularly on mud substrates between month 3 and 7 against *An. funestus* may be attributed to fluctuations in body size, a trait for which wing length is a standard indicator. Larger mosquitoes possess greater energy reserves, which can enhance their tolerance to chemical stressors and facilitate recovery from sublethal exposure [45]. These biological differences between mosquito cohorts throughout the testing period may have contributed to the observed variability. However, in this study, wing length was not measured on a monthly basis.

### 4.4. Substrate Effect on Efficacy

Wall materials such as mud, concrete, and wood can affect IRS residual efficacy, as absorption or adsorption into the substrate reduces the bioavailable dose for mosquito contact [46]. In this study, both IRS products performed poorly on mud surfaces, particularly against *An. funestus*, likely due to the high porosity of mud, which absorbs insecticide into the wall matrix and shortens residual activity [46]. In addition, the reduced efficacy of Sylando^®^ 240SC was also observed on concrete (pH 9–10). Alkaline pH can chemically degrade insecticides [44,48], although chlorfenapyr is reported to be relatively stable to hydrolysis at alkaline pH [49]. Its lower performance may therefore be more likely explained by adsorption and penetration into wall pores, which reduces surface availability for mosquito contact [50]. Emerging approaches such as porous silica particles, designed to maintain insecticide bioavailability on treated surfaces, are currently in trials [51].

### 4.5. Comparative Assessment of Bioassay Methods for Chlorfenapyr

#### 4.5.1. WHO Cone and Cylinder Bioassays

Cone and cylinder assays have long served as WHO standards due to their reproducibility, simplicity, and ability to harmonize results across laboratories and field sites [25,52,53]. However, both were designed for fast-acting neurotoxins and rely on brief, confined exposures. Such conditions are poorly suited to insecticides like chlorfenapyr, which require sustained activity for metabolic activation [6]. Confinement restricts movement, limiting uptake and underestimating efficacy, (Table 2) [2,18,32,54,55]. As a result, the WHO recommends overnight bottle assays for chlorfenapyr resistance monitoring with careful consideration of temperature and solvent [56].

#### 4.5.2. WHO Tunnel Bioassays

Tunnel assays introduced a more realistic environment, allowing mosquitoes to fly, seek hosts, and contact treated surfaces overnight, (Table 2) [57,58]. This design improved the evaluation of slow-acting chemistries, producing results more predictive of field outcomes than cone or cylinder tests. The tests are conducted overnight when metabolic enzymes that convert chlorfenapyr to tralopyril [59] are upregulated [8].

#### 4.5.3. Experimental Hut Trials

Experimental huts, developed in West and East Africa, remain the benchmark for realistic evaluation of vector control interventions [12,13]. They enable direct observation of entry, feeding, resting, and mortality under conditions close to household environments (Table 2). For chlorfenapyr, hut trials confirmed the efficacy that laboratory assays had underestimated [32,60] and link well to observations from clinical trials.

#### 4.5.4. Ifakara Ambient Chamber Test (I-ACT)

The I-ACT was developed to provide a more realistic testing environment by combining ecological realism with experimental control. It allows free-flying, overnight mosquito exposure in a defined volumetric space, under standardized conditions of surface type, application quality, temperature, and humidity (Table 2). For chlorfenapyr, I-ACT highlighted the importance of extended post-exposure mortality, validated efficacy against resistant strains, and enabled testing of species unavailable for hut trials. Compared with experimental huts, I-ACT offers the option to use a range of vector strains, and releasing high numbers of mosquitoes each night offers greater statistical power, consistency, operational feasibility and cost-effectiveness while still reflecting more natural mosquito behaviour.

#### 4.5.5. Recommendations

For more rigorous evaluation of IRS performance, future studies should consider including monthly assessment of mosquito physiological condition (wing length), wall substrate pH, and quarterly quantification of insecticide residue where methods exist. Similar studies across diverse settings are recommended due to differences in the chemical composition of concrete and mud in different settings. The development of standard “mud” and “cement” with specific parameters is recommended.

## 5. Conclusions

Sylando^®^ 240SC was demonstrated to be non-inferior to SumiShield^®^ 50WG against Afrotropical vectors at 72 h post exposure over a 12-month period. Cones and cylinders are useful for benchmarking neurotoxins but underestimate pro-insecticide chemistries. Tunnels have not been adapted for testing IRS and attempts in this lab have failed. Huts provide behavioural realism but usually need to be run for prolonged periods, may only have one or two target species, and sometimes face operational and reproducibility challenges. Each method has contributed to understanding insecticidal efficacy, but the evaluation of chlorfenapyr highlights their limitations when applied rigidly. I-ACT with MEH offers a platform for assessing novel, non-neurotoxic insecticides for IRS in controlled settings while still requiring confirmation in field settings.

## Figures and Tables

**Table 1:** Filter paper results for the chemical content analysis.

Test	Target Concentration	Actual Mean Dose Applied (Relative SD)
Chlorfenapyr content	250 mg/m^2^	230 mg/m^2^ (12.2%)
Clothianidin content	300 mg/m^2^	299 mg/m^2^ (30.5%)

**Table 2:** Vector control testing methods for slow-acting insecticides like chlorfenapyr.

Method	Purpose	Design and Conditions	Strengths	Limitations	Suitability for Chlorfenapyr
WHO Cone Bioassay	Lab-based efficacy testing	Mosquitoes confined to a treated surface for 3 min	Simple, reproducible, globally standardized	Restricts movement; unsuitable for metabolic or repellent insecticides	POOR Underestimates efficacy due to limited exposure and bioactivation
WHO Cylinder Bioassay	Resistance monitoring	Mosquitoes exposed to insecticide-treated paper in a cylinder	Standardized for resistance detection; easy to implement	Same confinement issues as cones; surfactant/paper inconsistencies	POOR Physical and chemical limitations affect chlorfenapyr performance
CDC Bottle Bioassay	Resistance monitoring	Mosquitoes exposed to insecticide-coated bottle at a discriminating concentration and time	Allows mosquito movement, flexible, standardized for resistance detection	Semi-artificial; limited behavioural realism, time-consuming if more than one mosquito strain is used	MODERATE Better than cones, and useful laboratory assay for IRS
WHO Tunnel Test	Semi-controlled behavioural assay	Mosquitoes fly through a tunnel to reach a host; exposure to treated netting	Simulates host-seeking behaviour; multiple endpoints (mortality, deterrence, feeding)	Still semi-artificial; limited exposure time	MODERATE Better than cones, and useful laboratory assay for ITNs, though undeveloped for IRS
Ifakara Ambient Chamber Test (I-ACT)	Controlled, semi-field evaluation	Large walk-in chamber mimicking a room; mosquitoes fly freely overnight	High realism, controlled environment, strong statistical power; multiple endpoints (mortality, feeding)	Requires infrastructure; relatively new method; uses laboratory-reared mosquitoes	GOOD Useful for exploring new slow-acting, metabolic non-repellent insecticides like chlorfenapyr
Experimental Hut Trials	Field-realistic efficacy testing	Free-flying mosquitoes interact with treated surfaces in a hut	Realistic behaviour with wild mosquitoes; multiple endpoints (mortality, feeding)	Operational variability; requires skilled personnel and equipment	EXCELLENT Captures effects of metabolic non-repellent insecticides like chlorfenapyr using wild mosquitoes that are highly active during host seeking

## Data Availability

The original contributions presented in this study are included in the article/Supplementary Material. Further inquiries can be directed to the corresponding author.

## Supplementary Materials

The following supporting information can be downloaded at https://doi.org/10.5281/zenodo.18541623, Table S1: Resistance profile of tested strains; 24 h mortality results: Q1-2023, Table S2: Non-inferiority of Sylando^®^ 240SC to SumiShield^®^ 50WG over 12 months with all substrates combined (concrete, wood and mud) of three Afrotropical vector strains (pyrethroid-susceptible *An. gambiae s.s.*, pyrethroid-resistant *An. arabiensis* and -resistant *An. funestus*). Figure S1: The median temperature and humidity inside the IACT and holding room during the experiment over 12 months of evaluation. Figure S2: Percentage mortality at 168 h post exposure to substrates sprayed with Sylando^®^ 240SC and SumiShield^®^ 50WG in I-ACT chamber assay over 12 months of evaluation: (**a**–**c**) susceptible strain *An. gambiae s.s.*; (**d**–**f**) pyrethroid-resistant *An. arabiensis*; (**g**–**i**) pyrethroid-resistant *funestus*; Figure S3: Percentage mortality at 168 h post exposure to substrates sprayed with Sylando^®^ 240SC and SumiShield^®^ 50WG in I-ACT chamber assay over 12 months of evaluation: (**a**–**c**) *Aedes aegypti*, fully pyrethroid-susceptible; (**d**–**f**) pyrethroid-resistant *Culex quinquefasciatus*; (**g**–**i**) pyrethroid-resistant *Ae. aegypti*.

## Abbreviations

The following abbreviations are used in this manuscript:

ATP	Adenosine Triphosphate
CFV	Control Flow Valve
DDT	Dichlorodiphenyltrichloroethane
IACT	Ifakara Ambient Chamber Test
IQR	Interquartile Range
IRS	Indoor Residual Spray
ITN	Insecticide Treated Nets
MEH	Miniature-Experimental Hut
NMCP	National Malaria Control Programmes
qRT-qPCR	Quantitative Reverse Transcription Polymerase Chain Reaction
RT-qPCR	Reverse Transcription Quantitative Polymerase Chain Reaction
RH	Relative Humidity
WHO	World Health Organization
WHO PQT/VCP	World Health Organization Prequalification Team—Vector Control Products
